# Serum biomarkers of neuroinflammation and blood-brain barrier leakage in amyotrophic lateral sclerosis

**DOI:** 10.1186/s12883-022-02730-1

**Published:** 2022-06-11

**Authors:** Maize C. Cao, Erin E. Cawston, Grace Chen, Collin Brooks, Jeroen Douwes, Dave McLean, E. Scott Graham, Mike Dragunow, Emma L. Scotter

**Affiliations:** 1 Department of Pharmacology and Clinical Pharmacology, University of Auckland, 85 Park Road, Auckland, 1023 New Zealand; 2 Centre for Brain Research, University of Auckland, Private Bag 92019, Auckland, 1142 New Zealand; 3 Centre for Public Health Research, Massey University, PO Box 75, Wellington, 6140 New Zealand; 4 Department of Molecular Medicine and Pathology, University of Auckland, 85 Park Road, Auckland, 1023 New Zealand

**Keywords:** Amyotrophic lateral sclerosis, Serum, Cytokine, Blood-brain barrier, Neuroinflammation

## Abstract

Amyotrophic lateral sclerosis (ALS) is an incurable and rapidly progressive neurological disorder. Biomarkers are critical to understanding disease causation, monitoring disease progression and assessing the efficacy of treatments. However, robust peripheral biomarkers are yet to be identified. Neuroinflammation and breakdown of the blood-brain barrier (BBB) are common to familial and sporadic ALS and may produce a unique biomarker signature in peripheral blood. Using cytometric bead array (*n* = 15 participants per group (ALS or control)) and proteome profiling (*n* = 6 participants per group (ALS or control)), we assessed a total of 106 serum cytokines, growth factors, and BBB breakdown markers in the serum of control and ALS participants. Further, primary human brain pericytes, which maintain the BBB, were used as a biosensor of inflammation following pre-treatment with ALS serum. Principal components analysis of all proteome profile data showed no clustering of control or ALS sera, and no individual serum proteins met the threshold for statistical difference between ALS and controls (adjusted *P* values). However, the 20 most changed proteins between control and ALS sera showed a medium effect size (Cohen’s d = 0.67) and cluster analysis of their levels together identified three sample subsets; control-only, mixed control-ALS, and ALS-only. These 20 proteins were predominantly pro-angiogenic and growth factors, including fractalkine, BDNF, EGF, PDGF, Dkk-1, MIF and angiopoietin-2. S100β, a protein highly concentrated in glial cells and therefore a marker of BBB leakage when found in blood, was unchanged in ALS serum, suggesting that serum protein profiles were reflective of peripheral rather than CNS biofluids. Finally, primary human brain pericytes remained proliferative and their secretome was unchanged by chronic exposure to ALS serum. Our exploratory study suggests that individual serum cytokine levels may not be robust biomarkers in small studies of ALS, but that larger studies using multiplexed analysis of pro-angiogenic and growth factors may identify a peripheral signature of ALS pathogenesis.

**Supplementary Information:**

The online version contains supplementary material available at 10.1186/s12883-022-02730-1.

## Introduction

Amyotrophic lateral sclerosis (ALS) is a debilitating condition characterized by the progressive degeneration of upper and lower motor neurons, and is usually fatal within 2–5 years [1, 2]. Frustratingly, for many people with ALS there is a delay of 1 year or more between symptom onset and diagnosis [3, 4]. A plethora of mechanisms contribute to neuronal damage in ALS, including impaired protein homeostasis, mitochondrial dysfunction, aberrant RNA metabolism and neuroinflammation [5–7]. The cause of disease is likely a combination of environmental and genetic risk factors [8, 9]. There is a clear need for effective diagnostic and treatment strategies for ALS, yet this heterogeneous etiology makes biomarker identification challenging.

Neurodegeneration in ALS is often accompanied by inflammation in the central nervous system (CNS), termed neuroinflammation. Indeed, neuroinflammation has been implicated in both sporadic and familial cases of ALS [10, 11]. This is characterized by the infiltration of immune cells from the periphery into the CNS and the activation of CNS-resident glial cells such as microglia and astrocytes. It is still undetermined whether neuroinflammation in ALS is a cause or consequence of motor neuron death, or both, but there is growing evidence that non-neuronal cells play critical roles in regulating motor neuron health [12, 13]. The inflammatory responses of these non-neuronal cells are highly complex and context-dependent, and may be either protective or pathogenic to motor neurons [14, 15].

The structure responsible for regulating immune cell infiltration into the CNS is the blood-brain barrier (BBB). The BBB strictly controls the transmission of both cells and molecules from blood to brain and vice versa. The BBB is enforced by several cell types including endothelial cells, astrocytes and pericytes; endothelial cells in particular are knitted together by tight junctions and exhibit limited transcellular transport, restricting passage both between and across cells. Pericytes are critical to BBB integrity by stabilizing endothelial tight junctions and suppressing transcytosis across endothelia [16–19]. We and others have shown that pericytes respond to pro-inflammatory stimuli by secreting a vast range of chemokines and cytokines, indicating that pericytes also have a role in mediating the inflammatory response [20, 21]. In ALS, there are cases in which the BBB is perturbed, as shown by the leakage of blood-derived proteins into the cerebrospinal fluid (CSF) [22–25] and brain parenchyma [25–27]. Among these, hemoglobin is toxic to motor neurons by inducing oxidative stress [28], but the serum component of ALS blood is currently believed to be non-cytotoxic to motor neurons [29]. Correlating with the presence of serum proteins in the CNS, there are large reductions observed in post-mortem tissue in the number of pericytes at the blood-spinal cord barrier (BSCB), and this is thought to contribute to the loss of microvascular integrity and eventual breakdown of the barrier [18, 27]. Blood-brain barrier leakage suggests that neuroinflammation in ALS may not be restricted to the CNS tissue, so blood factors may correlate with and predict disease progression. Supporting this notion, disease-related inflammatory changes detected in the blood often mirror those in CSF [30, 31].

Inflammatory cytokines previously shown to be mildly upregulated in ALS blood include CCL5 (RANTES), TGF-β1, IL-1β and IL-6, while IL-23, IL-8, TNF-α and its soluble receptors were moderately upregulated [30–36]. Meta-analysis of several of these smaller studies found other cytokines to be unchanged (MCP-1, IFN-γ, interleukins 2, 4, 5, 10, 17) [36]. However, conflicting findings, and a lack of information about the possible utility of *multiple* cytokine biomarkers in peripheral blood, led us to conduct multiplexed analysis of a panel of these cytokines. Also, given our experience in characterising the inflammatory responses of primary human brain pericytes to some of the implicated cytokines (IL-1β, TNFα) [21, 37, 38], we reasoned that if ALS serum indeed harbours increased levels of these or other cytokines, then by chronically exposing human brain pericytes to ALS sera we might use these cells as biosensors of inflammation. While endothelial cells are considered to be the major regulator of the BBB, we have previously found both cell types grown from human brain to be responsive to inflammation [37] and cultured pericyte yield is far higher. Brain pericytes are not in direct contact with the blood in the healthy state, but may be exposed to blood during BBB breakdown, and this may be relevant to their degeneration in ALS [18, 27].

In this exploratory study, rather than examining small numbers of individual proteins as previous studies have, we compared ALS and control serum levels of two panels totalling 106 different cytokines and growth factors. We sought to determine if there were differences in overall serum composition that might support previous findings of peripheral inflammation, and which might be exploited as biomarkers. We also applied the sera over live pericyte cells, using them as a novel biosensor to determine whether constituents of the serum altered their health or inflammatory responses. Finally, we measured levels of S100β in ALS and control sera to investigate the integrity of the BBB. Together, these experiments aimed to assess neuroinflammatory or BBB leakage markers in ALS blood sera that may have diagnostic/prognostic potential or shed light on the pathogenesis of disease.

## Methods

### Subjects and ethics approval

Fifteen ALS (10 males and 5 females, mean age 67.3 ± 10.4 years (SD)) and 15 healthy control participants (10 males and 5 females, mean age 75.9 ± 6.3 years (SD)) took part in this study. All participants had previously been involved in a case-control study examining occupational exposures and motor neuron disease [39]. Inclusion criterion for cases was based on a primary or secondary diagnosis of at least “probable” motor neuron disease by neurologists applying revised El Escorial, Awaji or other clinical criteria [40, 41]. For every case, one control was randomly selected from the New Zealand Electoral Roll (2008), frequency-matched by sex. Controls with a neurodegenerative disease were excluded. To be eligible for blood collection, controls and cases needed to be alive in October 2015 and be based in the Wellington region, and give informed consent to blood collection by a visiting nurse (participant details in Tables S1a and S1b). Ethics approval was granted by the New Zealand Multi-Region Ethics Committee (ref. MEC/12/01/005/AM03) and methods were performed in accordance with the approved guidelines and regulations. The demographics and co-morbidities of the ALS and control groups were well balanced, ensuring homogeneity when comparing the two groups (Table S1c). Human brain pericytes used in this study were derived from biopsies of patients with drug-resistant epilepsy and obtained with informed donor consent. All experiments conducted involving human tissue were approved by the Northern Regional Ethics Committee (New Zealand) and carried out in accordance with the approved guidelines.

### Collection and processing of blood serum

Approximately 10 mL of whole blood was collected from each subject into a BD Vacutainer Serum Tube (#367895, BD Diagnostics, NJ, USA) and left to clot at room temperature for 60 minutes. Clotted serum was then centrifuged at 2000 x *g* for 10 minutes at 20 °C and placed on ice. Serum was removed into a fresh 15 mL tube, and 250 μL aliquots were stored in 1.4 mL micronic cryovials at − 80 °C until assayed. All experiments were carried out blinded as to the disease status of the donor.

### Serum cytokine detection by cytometric bead array

Cytokine concentrations were measured directly in serum from control and ALS participants, and also in conditioned media samples from sera- then cytokine-stimulated pericytes. Serum was centrifuged at 2000 x *g* for 10 minutes, and conditioned media at 180 x *g* for 5 min, to remove cells or debris. Samples were then assayed using CBA (BD Biosciences, CA, USA). Sera were used undiluted (neat) to measure the concentrations of human cytokines IL-6, CXCL8 (IL-8), CCL5 (RANTES), CCL2 (MCP-1), CXCL10 (IP-10) and CX3CL1 (fractalkine); and sera were diluted 1:50 in assay diluent to measure the concentrations of human soluble CD54 (sICAM-1) and soluble CD106 (sVCAM-1) (Product details in Table S2; Optimization of serum dilutions in Fig. S1). All CBA samples were run on an Accuri C6 flow cytometer (BD Biosciences, CA, USA). Data was quantified using FCAP-array software (version 3.1) (BD Biosciences, CA, USA) to convert fluorescence intensity values to concentrations. Cytokine concentrations for conditioned media were then normalized to cell number. There were no associations between serum cytokine concentrations and participant age (Fig. S2) or duration of disease (Fig. S3) so these factors were not considered to be confounders.

### Serum cytokine and growth factor detection by proteome profiler array

Having quantified by CBA a panel of cytokines known to be implicated in ALS, we tested whether previously untested cytokines and growth factors may be altered in ALS participant sera. Six ALS and six control sera samples were assayed using the Proteome Profiler Human XL Cytokine Array (#ARY022, R&D Systems, MN, USA), which determines the relative levels of 105 human cytokines and growth factors (as listed in Table S3), according to the manufacturer’s directions. Samples were selected such that there were three males and three females in each group, cases were age matched (ALS group mean age = 67.5 ± 10.3 years, control group mean age = 72.7 ± 6.6 years), and males and females in the ALS group had similar durations of disease (ALS male mean duration = 483 days, ALS female mean duration = 762 days). Membranes were imaged using LI-COR Odyssey Fc imaging system and quantified using Image Studio Lite Ver. 5.0 software (LI-COR Biosciences, NE, USA).

### Pericyte culture

Brain tissue from the middle temporal gyrus was processed for the isolation and culture of mixed glial cultures initially containing pericytes, astrocytes and microglia [42]. Cells were maintained until confluent in DMEM/F-12 with 10% fetal bovine serum (source), 100 U/mL penicillin, 100 μg/mL streptomycin, and 0.292 mg/mL glutamine (Gibco, CA, USA) and then passaged by harvesting with 0.25% Trypsin-EDTA (Gibco, CA, USA). Early passages up to passage 5 contained a mixed glial culture of pericytes, microglia and astrocytes [21, 38]. Due to the proliferation of pericytes, which overgrow the non-proliferative glial cells, later passage cultures exclusively contain pericytes [37, 38, 43]. The cells used in this study were passage 6 or greater. Cells were plated at 15,000 cells per cm^2^ in 96-well plates.

### Serum and pro-inflammatory compound treatments of pericytes

ALS or control sera were diluted to final 20% in DMEM/F-12 with 100 U/mL penicillin, 100 μg/mL streptomycin, and 0.292 mg/mL glutamine (Gibco, CA, USA) and incubated with human brain pericytes. Sera were removed after 96 hours and cells washed with serum-free DMEM/F12 before incubation with serum-free DMEM/F12 containing 0.05 ng/mL interleukin 1 beta (IL-1β), 0.1 ng/mL interferon gamma (IFN-γ), or 0.25 ng/mL tumour necrosis factor alpha (TNF-α), each also containing final 0.001% bovine serum albumin, or vehicle (0.001% bovine serum albumin) for 8 hours or 24 hours. Concentrations of pro-inflammatory cytokines were selected based on estimated EC50 values [21]. Plates of pericytes subjected to 8-hour cytokine treatment were fixed and stained using immunocytochemistry. Conditioned media was collected from both 8 and 24-hour samples for detection of cumulative secreted cytokine (IL-6, IL-8, RANTES, MCP-1, IP-10, fractalkine, sICAM-1 and sVCAM-1) by CBA, as described above.

### Immunocytochemistry

Cells were fixed in 4% paraformaldehyde for 10 minutes and then washed in phosphate buffered saline with 0.1% Triton X-100 (PBS-T). Primary antibodies (Table S4) were diluted in immunobuffer (PBS containing 1% goat or donkey serum, 0.2% Triton X-100 and 0.04% thimerosal) and incubated with cells overnight at 4 °C. Cells were washed in PBS-T and then incubated with the appropriate fluorescent secondary antibodies (Table S4) for 4 hours at room temperature. Cells were washed again, and cell nuclei were counterstained with Hoechst 33258 (Sigma-Aldrich, MO, USA) for 20 min. Images were acquired at 10X magnification on an ImageXpress Micro XLS automated fluorescence microscope (Molecular Devices, CA, USA). Integrated intensity measures (VCAM-1) and percentage of total cells that stained positively (MCP-1) were quantified using the MultiWavelength Cell Scoring module within MetaXpress software (Molecular Devices, CA, USA).

### S100β ELISA assay

Serum samples from all ALS and control participant samples were analyzed undiluted, using the Human S100β ELISA kit (#EZHS100B-33 K, EMD Millipore, MA, USA) according to manufacturer’s directions. Absorbance was read at 450 nm (test) and 590 nm (light scatter) with a FLUOstar Optima (BMG LABTECH, Offenburg, Germany) to give A_450 nm_-A_590 nm_. The standard curve was fitted to a sigmoidal 4-parameter logistic (4PL) equation and concentrations of samples were calculated with a 4PL function (Fig. S4). The sensitivity limit of the assay was 2.7 pg/mL.

### Statistical analysis

Statistical analysis of serum CBA data was performed using GraphPad Prism 8.0 software (La Jolla CA, USA). Outliers were removed by the ROUT method, Q = 1%. Principal component analysis and clustering analysis for proteome profiler data were carried out using R software. Serum and pericyte conditioned media cytokine and growth factor concentrations were analyzed using Mann Whitney U-tests, with *P* values then adjusted for false discovery rate by Benjamini-Hochberg method. Statistical significance was considered to be adjusted *P* value < 0.05. Post-hoc power calculations were determined with the ‘pwr’ package in R software and the effect size was determined with Cohen’s d formula.

## Results

### Cluster analysis of the serum proteome partially segregates ALS and control

**Fig. 1:**
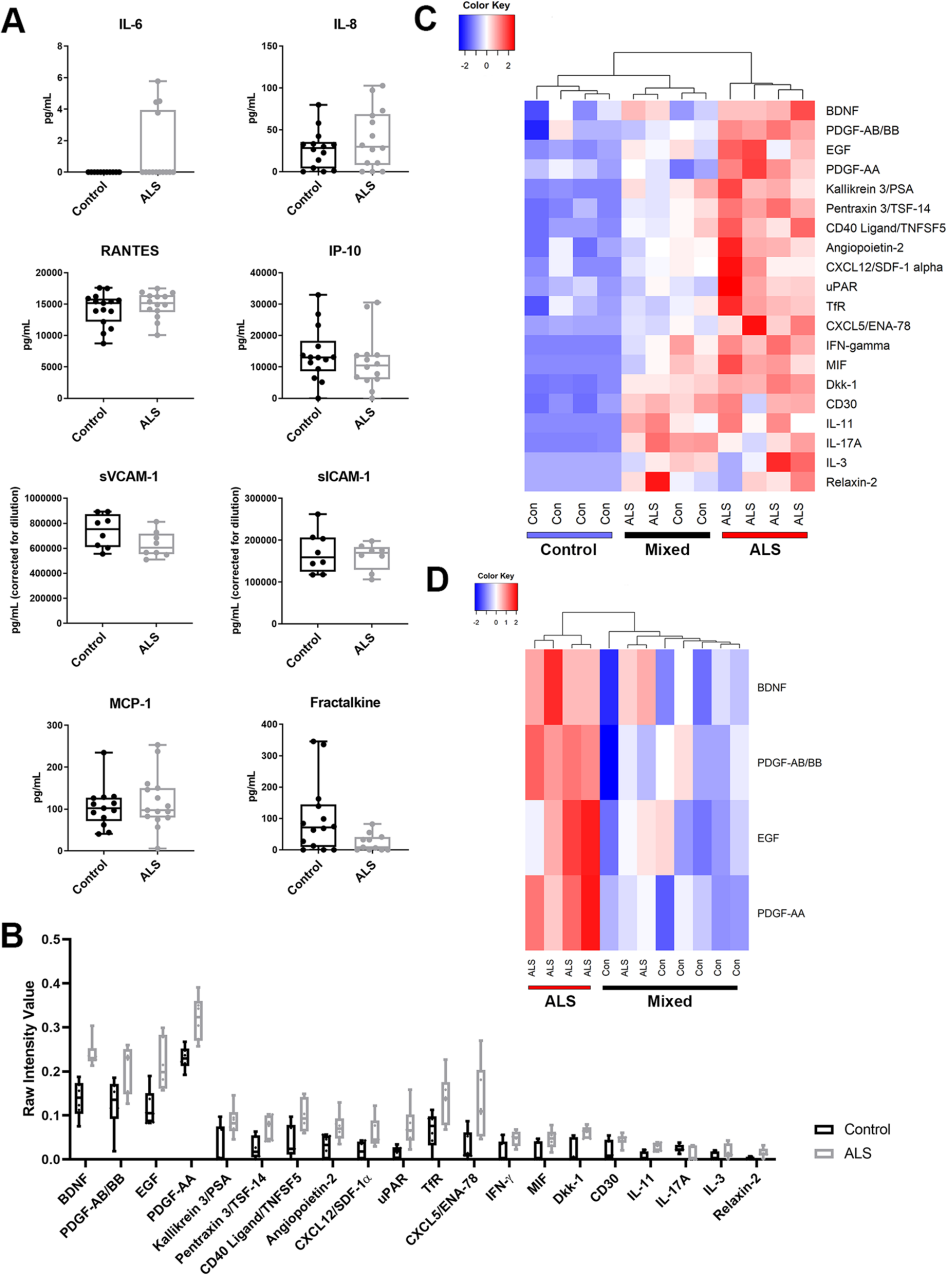
Clustered proteomic analysis of sera partially segregates ALS and control. **A** Cytometric bead array analysis of participant blood sera. IL-6, IL-8, RANTES, MCP-1, IP-10, fractalkine in control (*n* = 15) and ALS (*n* = 15) sera and sICAM-1 and sVCAM-1 in control (*n* = 8) and ALS (*n* = 8) sera. Boxes, 25th -75th percentiles; whiskers, minima and maxima. Statistical significance determined by Mann Whitney test. **B** Proteome profiler analysis of an extended panel of 105 cytokines in control (*n* = 6) and ALS (*n* = 6) participant blood sera. Raw intensity normalized to reference spot intensity of each membrane. Proteins (*n* = 20) with the greatest difference between ALS/controls shown. Boxes, 25th -75th percentiles; whiskers, minima and maxima*.* Statistical analysis determined by Mann-Whitney test. **C** Heat map shows unsupervised hierarchical clustering of the listed cytokines. **D** Unsupervised hierarchical clustering of growth factors BDNF, EGF and PDGF-AB/BB/AA

To test whether there were inflammatory changes in ALS compared to control participant sera, the sera were first tested using CBA for a panel of 8 cytokines that are key markers of inflammatory processes – IL-6, IL-8, RANTES, MCP-1, IP-10, fractalkine, sICAM-1 and sVCAM-1. The serum pro-inflammatory cytokine concentrations in control and ALS groups showed similar distribution. There was a decrease in fractalkine in the sera of ALS participants (raw *P* < 0.05), however the adjusted *P* value was not significant, and no changes were evident in the other cytokines examined (Fig. 1 A).

We next assayed a subset of 6 ALS and 6 control sera samples using the Proteome Profiler Human XL Cytokine Array, which determines the relative levels of 105 human cytokines and growth factors. These included pro-inflammatory cytokines within our CBA panel (IL-6, IL-8, MCP-1, IP-10, RANTES, ICAM-1 and VCAM-1), and factors not in our cytokine CBA panel but implicated in ALS (MMP9, angiogenin, angiopoietin-2, VEGF, uPAR) [44–47]. Principal components analysis of all proteome profile data showed no clustering of control or ALS sera samples (Fig. S5). Significant differences were observed between control and ALS sera in 20 factors (BDNF, PDGF-AB/BB, EGF, PDGF-AA, kallikrein 3, pentraxin 3, CD40 ligand, angiopoietin-2, CXCL12, uPAR, TfR, CXCL5, IFN-γ, MIF, Dkk-1, CD30, IL-11, IL-17A, IL-3, relaxin-2) (raw *P* < 0.05) but, as with fractalkine, the adjusted *P* value was not significant (Fig. 1 B). We then tested whether clusters of multiple factors could discriminate ALS from control sera. When serum levels of the 20 factors identified above were compared between ALS and controls by unsupervised hierarchical clustering, we observed clustering (Fig. 1 C). Segregation remained incomplete, with three subsets emerging; a control-only subset, a mixed control-ALS subset, and an ALS-only subset. The proteins most strongly associated with these clustered cytokines included the growth factors BDNF, PDGF-AB/BB, EGF and PDGF-AA. Unsupervised hierarchical clustering based on these four factors alone also did not fully segregate the control and ALS samples (Fig. 1 D).

### ALS serum pre-treatment did not influence the pericyte secretome or cell number in response to pro-inflammatory compounds

**Fig. 2:**
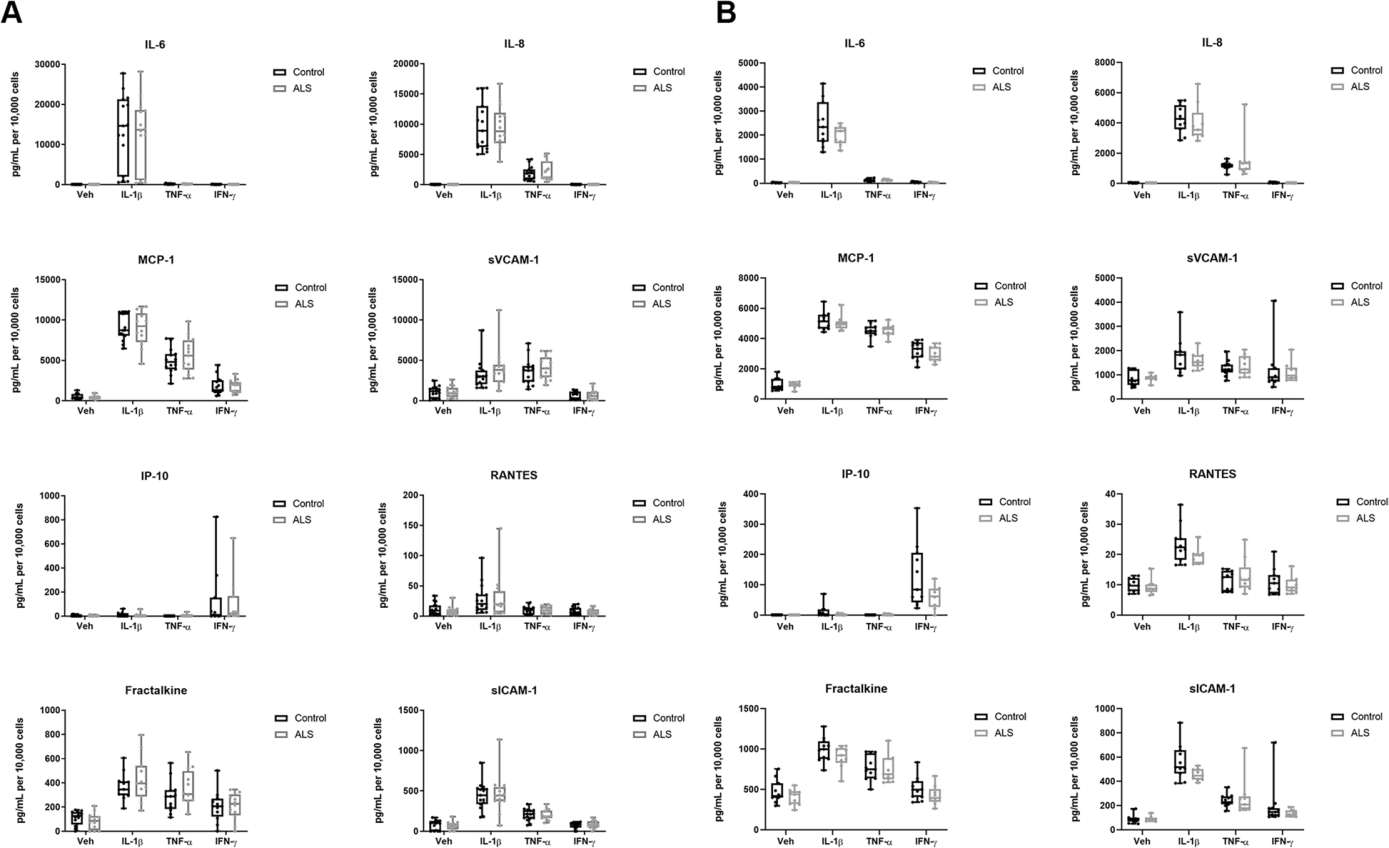
ALS serum pre-treatment did not influence the pericyte secretome in response to pro-inflammatory compounds. **A** Cytometric bead array analysis of selected cytokines secreted by primary human brain pericytes pre-treated with control (black, *n* = 15) or ALS (grey, *n* = 15) participant sera, then exposed to pro-inflammatory stimuli for 24 hours. Boxes, 25th -75th percentiles; whiskers, minima and maxima. Statistical analysis between ALS and control determined by multiple Mann-Whitney tests. **B** As per (**A**) but exposed to pro-inflammatory stimuli for 8 hours

**Fig. 3:**
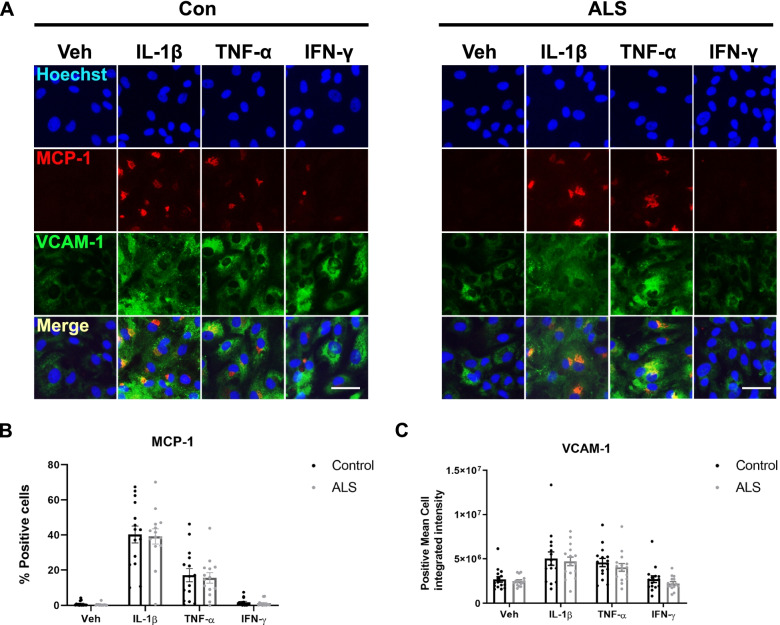
ALS serum pre-treatment did not influence pericyte intracellular expression of MCP-1 or VCAM-1 in response to pro-inflammatory compounds. **A** Representative images of ALS or control serum pre-treated pericytes 8 hours after vehicle or different pro-inflammatory stimuli were applied (IL-1β, IFN-γ or TNF-α). Scale bar = 50 μm. Inflammatory response is represented by an upregulation of MCP-1 and VCAM-1. **B** Quantification of percentage cells positive for MCP-1, mean +/− SEM. **C** Quantification of mean integrated intensity of VCAM-1 per positive cell, mean +/− SEM

**Fig. 4:**
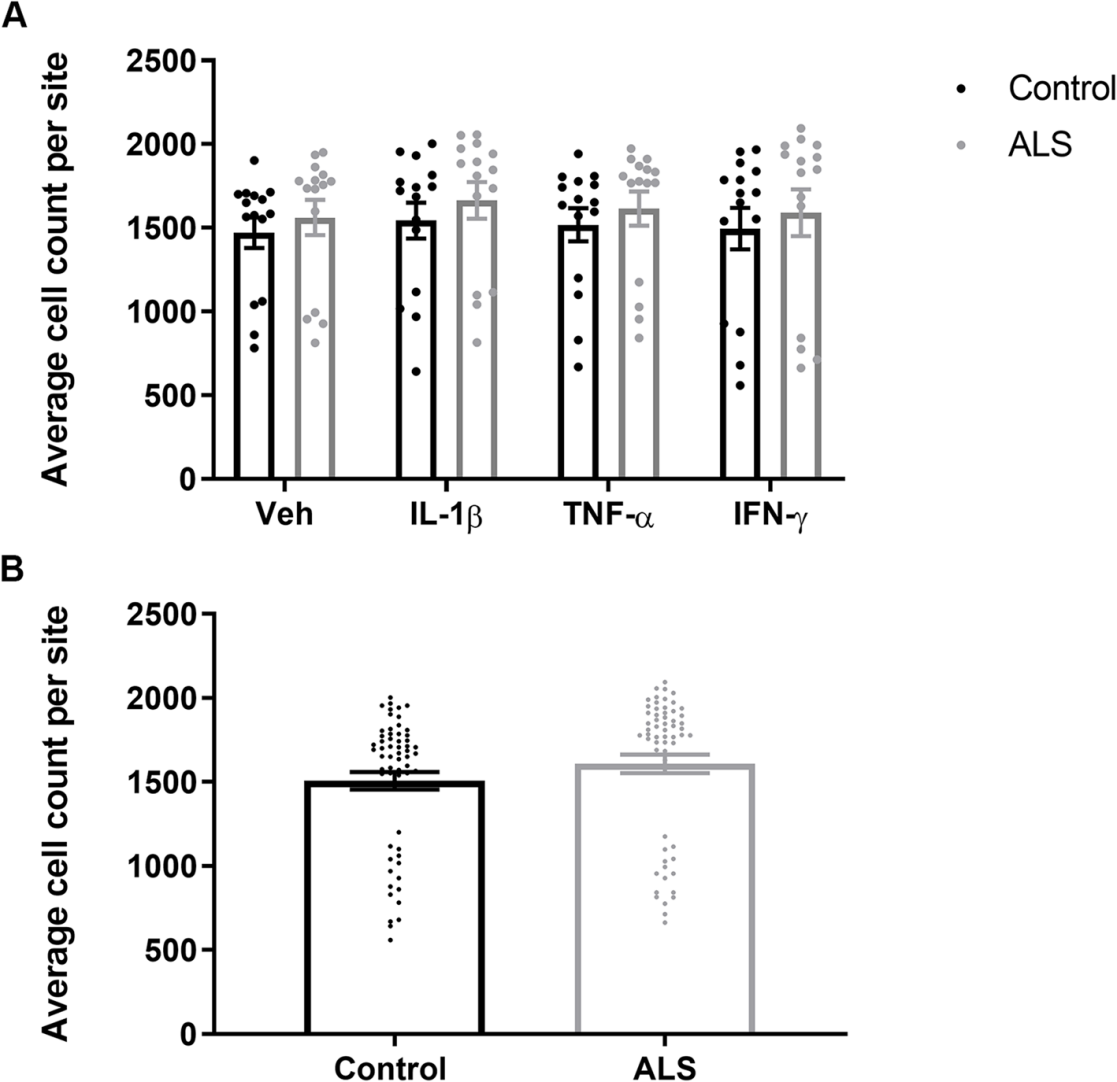
ALS serum pre-treatment did not significantly alter pericyte cell number. **A** There was a consistent but non-significant increase in ALS serum pre-treated cell nuclei observed across all pro-inflammatory stimuli and vehicle. Values are average cell counts per 10X magnification image site (equivalent to 1393.2 × 1393.2 μm). Two sites were imaged per well for each of the 15 ALS and 15 control sera-treated cell samples across all pro-inflammatory stimuli conditions and vehicle. Results are plotted as mean +/− SEM. **B** Overall combined cell nuclei count (from all treatments). Statistical analysis determined by Mann-Whitney test

We next tested whether chronic exposure of primary human brain pericytes to ALS participant sera (for 96 hours) altered their subsequent response to pro-inflammatory triggers IL-1β, IFN-γ or TNF-α, in terms of cytokine intracellular expression (at 8 hours) or secretion (at 8 and 24 hours). The secretome of serum-pre-treated human brain pericytes (96 hours) subjected to a pro-inflammatory stimulus for an additional 8 hours (Fig. 2 B) or 24 hours (Fig. 2 A) differed depending upon the stimulus, but not the source of the serum (control versus ALS participant). Secretome data for selected cytokines MCP-1 and VCAM1 was corroborated by intracellular staining (Fig. 3 A); ALS serum pre-treatment did not influence intracellular expression of MCP-1 or VCAM-1 by pericytes in response to vehicle or pro-inflammatory compounds (Fig. 3 B-C). Of the growth factors found to be elevated in ALS participant sera (BDNF, PDGF-AB/BB, EGF and PDGF-AA), PDGF-BB is well known to promote the growth of human brain pericytes [48–50]. We therefore analyzed whether the chronic (96 hour) pre-treatment with control or ALS sera differentially affected pericyte cell proliferation by analyzing nuclear counts of the serum-pre-treated vehicle or pro-inflammatory stimuli-treated cells described above. Average cell number per site imaged was found to be consistently higher when pre-treated with ALS serum, however this effect was not significant either when analyzed according to pro-inflammatory stimulus (Fig. 4 A) or when these stimuli were pooled (Fig. 4 B).

### S100β levels are not different between ALS and control sera

**Fig. 5:**
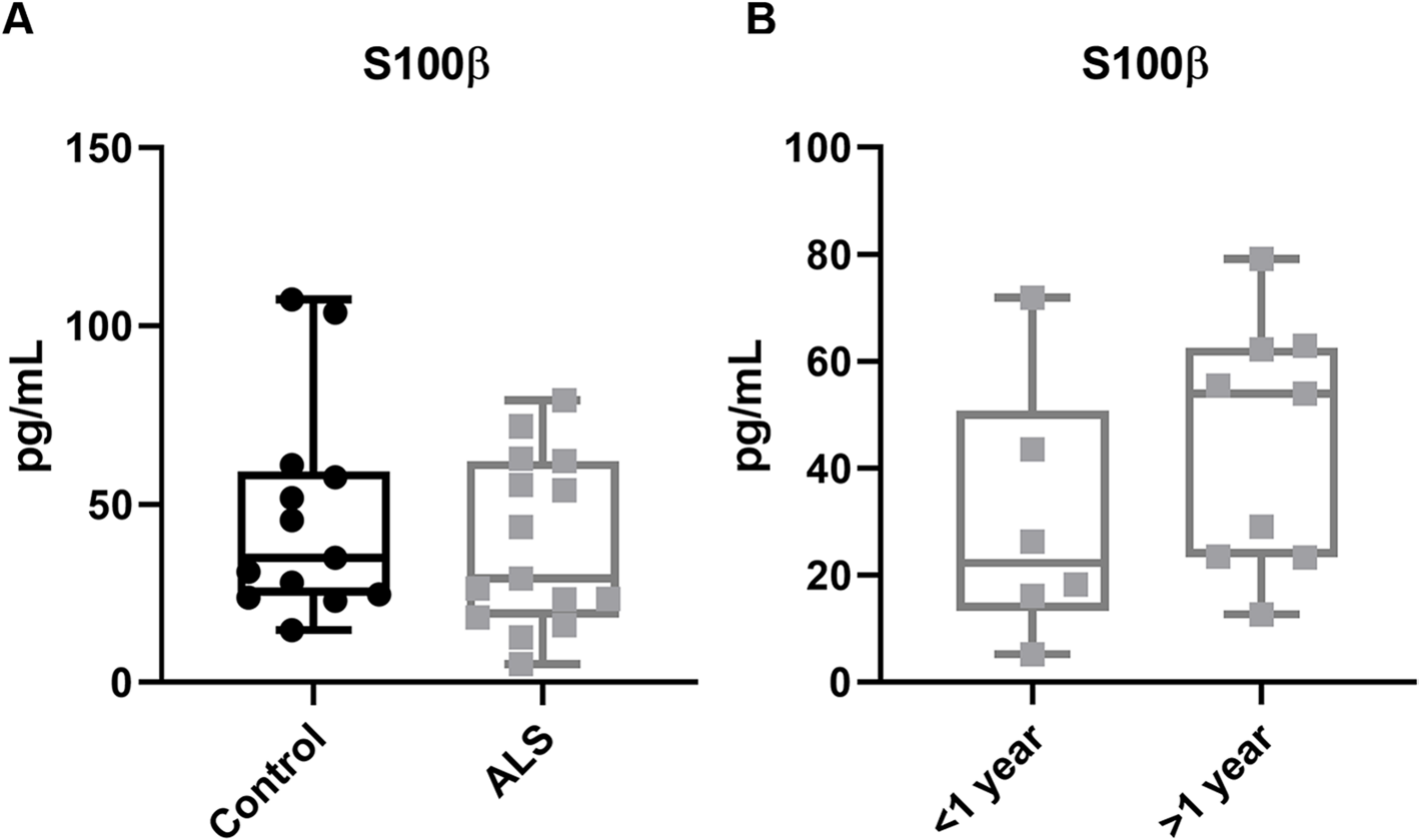
S100β levels in ALS sera are not different from control sera. **A** S100β levels detected in sera showed no difference between ALS and control groups. **B** ALS sera samples separated on the basis of disease duration. Results are plotted as mean +/− SEM and each dot represents the average concentration from one serum sample

We lastly tested the fidelity of the blood-brain barrier by measuring the serum levels of the astrocytic protein S100β, a widely used marker of BBB leakage in serum [51–53]. No difference was observed in S100β levels between ALS and control sera (Fig. 5 A). Nor was S100β altered with disease progression, with no significant difference in S100β concentrations between ALS sera from participants less than 1 year post-diagnosis versus greater than 1 year post-diagnosis (Fig. 5 B).

## Discussion

The pathogenesis of ALS is widely accepted to involve neuroinflammation relating to astrocyte and microglial activation [11, 54–58], and BBB leakage evidenced by loss of pericytes and parenchymal accumulation of serum components [18]. When the barrier between blood and nervous tissue is disrupted experimentally, inflammatory markers can be shared between the nervous tissue and blood [51, 59, 60], but it is unknown to what extent peripheral markers reflect CNS inflammatory state in ALS. In this study we found no significant changes in individual cytokines between control and ALS blood serum, which may be due to our small sample size, but found trends including decreased levels of fractalkine and increased vascular growth/ angiogenic factors. Whether these trends are driven by peripheral or central inflammation is unclear, however they may yet have predictive value as biomarkers if multiplexed.

Several of the serum factors that trended towards change in ALS serum, including fractalkine, BDNF, EGF, PDGF, Dkk-1, MIF, and uPAR, have pro-angiogenic effects [61–67]. Abnormal angiogenesis may occur in ALS according to evidence from postmortem tissue, in which increased microvascular density and accumulation of collagen fibers in the spinal cord perivascular space suggest compensation is occurring to maintain vascular integrity [68, 69]. The pro-angiogenic soluble uPAR is also increased in ALS patient spinal cord [47]. Angiogenic changes in ALS are not necessarily drivers of pathogenesis, and indeed ample evidence suggests that angiogenic changes in blood are a response to respiratory decline and hypoxia [70]. In line with this, angiopoetin-2 is strongly associated with acute respiratory distress [71, 72], and is present in hypoxemic ALS patients [45, 46]. Similarly, it has been proposed that the upregulation of IL-6 and TNF-α in ALS sera is a consequence of peripheral hypoxia rather than neurological decline [73]. Thus, changes in these serum factors likely relate more to peripheral inflammation, including dysregulated angiogenesis induced by hypoxia, than to central neuroinflammatory processes. This is supported by our finding, and that of others [74], that ALS patient sera did not have increased levels of the blood-brain barrier leakage marker S100β. Although elevated peripheral S100β levels are not always determinative of blood-brain barrier break-down [75], the lack of change in S100β between control and ALS sera indicates that CNS proteins in these donors remain partitioned from the periphery. Components of the CSF can drain into the circulation through the arachnoid villi via non-selective diffusion, so it is also possible that these serum factors derive from CSF [76].

Regardless of its origins, a distinct peripheral signature of ALS could be exploited as a diagnostic biomarker. A peripheral readout of early hypoxic changes due to sub-clinical respiratory decline could greatly assist with diagnosis [70]. For serum protein levels to be effective as biomarkers, clustering should be observed between disease subgroups and/or between disease and non-disease states. Combining data from the 20 most different serum factors identified by proteome profiling, with a medium effect size (Cohen’s d = 0.67), we could only partially discriminate control from ALS cases. The effect size was limited by the substantial inter-individual variability in individual serum protein levels independent of disease status, which likely reflects known individual variability in immune responsiveness [15]. Individual blood protein levels change with age, exercise, disease progression rate, stage of disease, and effects of disease such as hypoxia, as mentioned [10, 34, 73, 77, 78]. Additionally, these changes are not always chronic. Transient increases in HLA-DR, CD11c, and CX3CR1 (fractalkine receptor) have been observed in ALS as the result of acute myeloid expansion [78]. Such variability necessitates a larger sample size to detect disease-specific effects; post hoc power calculation based on this exploratory study shows that sample sizes greater than 37 per group should be used for multiple testing of a similar number (~ 20) of biomarkers with similar disease-specific difference to what we have identified here.

We further characterized ALS sera in terms of the ability to modify the activity of human brain pericytes. Pericytes are among the first responders to peripheral inflammation [79], and a recent study found that perivascular cells undergo significant gene changes that precede the ALS symptomatic stage [80]. Thus, pericytes have the potential to act as a biosensor of specific inflammatory mediators. In brain endothelial cells, the transcriptome is altered by age-related changes in the circulatory environment [81], and the endothelial cell secretome is altered by ALS plasma [82] indicating brain pericytes could also be a sensitive sensor to circulatory changes. Pericytes also proliferate in response to PDGF-BB, through interaction with the pericyte-enriched PDGF receptor β [83, 84]. However, chronic pre-treatment of human brain pericytes with either control or ALS sera altered neither cytokine secretion by stimulated pericytes nor pericyte proliferation. This suggests that the higher levels of PDGF-AB/BB and PDGF-AA measured in ALS sera by proteome profiling remain below the concentration range that stimulates pericyte proliferation (0.1–100 ng/mL) [84]. Our data suggest that the loss of pericytes in ALS [18, 27, 68, 69] is unlikely to be related to exposure to serum-derived proteins.

## Conclusion

In our small cohort of ALS and control cases, we did not detect significant differences in blood sera in any of 106 individual cytokines or growth factors, however a panel of 20 factors enriched for pro-angiogenic and growth factor function partially segregated ALS from control cases. The BBB leakage marker S100β was unchanged in ALS serum. While there are clear roles for neuroinflammation and BBB dysfunction in ALS pathogenesis, we suggest that a signature of disease identified in serum could be peripheral rather than central in origin, and comprise pro-angiogenic factors and growth factors rather than cytokines. Multiplexed analyses of larger numbers of samples (> 35 per group) would be required to exploit this peripheral signature as a biomarker.

## Supplementary Information

**Additional file 1.**

## Data Availability

All data generated or analysed during this study are included in this published article [and its supplementary information files].

## Abbreviations

4PL: 4 parameter logistic
ALS: Amyotrophic lateral sclerosis
BBB: Blood-brain barrier
BDNF: Brain-derived neurotrophic factor
BSCB: Blood-spinal cord barrier
CBA: Cytometric bead array
CCL5/RANTES: Chemokine ligand 5/ regulated upon activation, normal T cell expressed, and secreted
CD106/VCAM-1: Cluster of differentiation 106/ vascular cell adhesion molecule 1
CD30: Cluster of differentiation 30
CD40: Cluster of differentiation 40
CD54/ICAM-1: Cluster of differentiation 54/ intercellular adhesion molecule 1
CNS: Central nervous system
CSF: Cerebrospinal fluid
CX3CL1: C-X3-C Motif Chemokine Ligand 1 (fractalkine)
CXCL12: C-X-C motif chemokine 12
CXCL5: C-X-C motif chemokine 5
Dkk-1: Dickkopf-related protein 1
DMEM/F12: Dulbecco’s Modified Eagle Medium/Nutrient Mixture F-12)
EC50: Half maximal effective concentration
EDTA: Ethylenediamine tetraacetic acid
EGF: Epidermal growth factor
ELISA: Enzyme-linked immunosorbent assay
IFN-γ: Interferon gamma
IL-11: Interleukin 11
IL-17A: Interleukin 17A
IL-1β: Interleukin 1 beta
IL-3: Interleukin 3
IL-6: Interleukin 6
IL-8/CXCL8: Interleukin 8/ C-X-C motif chemokine ligand 8
IP-10/CXCL10: Interferon gamma-induced protein 10/ C-X-C motif chemokine 10
MCP-1/CCL2: Monocyte chemoattractant protein 1/ C-C motif chemokine ligand 2
MIF: Macrophage migration inhibitory factor
MMP9: Matrix metallopeptidase 9
PBS: Phosphate-buffered saline
PBS-T: Phosphate-buffered saline with Triton X-100
PDGF: Platelet-derived growth factor
S100β: S100 Calcium Binding Protein B
sICAM-1: Soluble intercellular adhesion molecule 1
sVCAM-1: Soluble vascular cell adhesion molecule 1
TfR: Transferrin receptor
TGF- β1: Transforming growth factor beta 1
TNF-α: Tumour necrosis factor alpha
uPAR: Urokinase receptor
VEGF: Vascular endothelial growth factor
