# Supplementary material for: Serum biomarkers of neuroinflammation and blood-brain barrier leakage in amyotrophic lateral sclerosis
Source: BMC Neurol. 2022 Jun 11;22:216. doi: 10.1186/s12883-022-02730-1 (PMC9188104; doi:10.1186/s12883-022-02730-1)
Supplement: Supplementary file 1 — Additional file 1. [file 12883_2022_2730_MOESM1_ESM.docx]

**Serum biomarkers of neuroinflammation and blood-brain barrier leakage in Amyotrophic Lateral Sclerosis**

**Supplementary Tables and Figures**

**Authors**

Maize C. Cao^1^, Erin E. Cawston^1^, Grace Chen^2^, Collin Brooks^2^, Jeroen Douwes^2^, Dave McLean^2^, E. Scott Graham^3^, Mike Dragunow^1*^, Emma L. Scotter^1*^

^1^ Department of Pharmacology and Centre for Brain Research, University of Auckland, 85 Park Road, Auckland 1023, New Zealand

^2^ Centre for Public Health Research, Massey University, PO Box 75, Wellington 6140, New Zealand

^3^ Department of Molecular Medicine and Pathology and Centre for Brain Research, University of Auckland, 85 Park Road, Auckland 1023, New Zealand

*Corresponding authors

Email [m.dragunow@auckland.ac.nz](mailto:m.dragunow@auckland.ac.nz).

Email [emma.scotter@auckland.ac.nz](mailto:emma.scotter@auckland.ac.nz).

**Supplementary Table 1a. Patient Clinical Details**

| **No.** | **Unique ID** | **Sex** | **Age**  **at sample collection (y)** | **Age**  **of onset (y)** | **Disease duration* (days)** | **Diagnostic delay** | **Subtype** | **Sporadic or familial** | **Site of onset** | **BMI** | **Highest education** | **Co-morbidities** |
| --- | --- | --- | --- | --- | --- | --- | --- | --- | --- | --- | --- | --- |
| 1 | MND003 | M | 81 | 75 | 460 | 4 years | ALS | Sporadic | Upper limbs | 24.3 | Undergraduate | Prostate cancer, hypertension |
| 2 | MND005^#^ | F | 71 | 62 | 2255 | 2 years | ALS | Sporadic | Bulbar | 22.1 | Undergraduate | Hypertension, palpitations |
| 3 | MND006^#^ | M | 61 | 58 | 647 | 1 year | ALS | Sporadic | Lower limbs | 24.8 | Undergraduate | Hypertension |
| 4 | MND007 | M | 59 | 58 | 259 | 5 months | ALS | Sporadic | Upper limbs | 21.2 | Technical/trade school | Hypertension |
| 5 | MND009^#^ | M | 64 | 60 | 502 | 2 years | ALS (bulbar onset) | Sporadic | Bulbar | 32 | Secondary school | Diabetes, raised cholesterol, hypertension |
| 6 | MND011 | M | 83 | 57 | 452 | 24 years | ALS (probable) | Sporadic | Lower limbs | 34.5 | Secondary school | Previous heart attack, raised cholesterol, hypertension |
| 7 | MND013 | M | 54 | 52 | 360 | 5 months | ALS | Sporadic | Lower limbs | 28.7 | Technical/trade school | Diabetes, raised cholesterol, hypertension |
| 8 | MND015 | M | 68 | 67 | 216 | 7-8 months | ALS | Sporadic** | Lower limbs | 31.5 | Postgraduate | Raised cholesterol |
| 9 | MND017^#^ | F | 82 | 76 | 840 | 3 years | Primary lateral sclerosis*** | Sporadic | Bulbar | 25.3 | Technical/trade school | None |
| 10 | MND021 | M | 73 | 71 | 469 | 9-10 months | ALS | Sporadic | Lower limbs | 29.4 | Undergraduate | Raised cholesterol, hypertension |
| 11 | MND022^#^ | M | 53 | 49 | 1329 | 7-8 months | ALS | Sporadic | Lower limbs | 25.2 | Undergraduate | Raised cholesterol |
| 12 | MND025 | F | 59 | 57 | 92 | 1.5 years | ALS | Sporadic**** | Lower limbs | 24.5 | Secondary school | Hypertension |
| 13 | MND026^#^ | F | 74 | Undetermined | 453 | Undetermined | ALS | Sporadic | Bulbar | 15.4 | Secondary school | None |
| 14 | MND029 | M | 76 | 75 | 132 | 5 months | ALS | Sporadic** | Upper limbs | 24.8 | Undergraduate | Raised cholesterol |
| 15 | MND030 | F | 52 | 51 | 172 | 5 months | ALS | Sporadic | Bulbar | 23.4 | Postgraduate | None |

^#^ Subject sample used for proteome profile array analysis

* Diagnosis to sample collection

**Mother had dementia

***Although clinically distinct from ALS, PLS was included due to mechanistic overlap between ALS and PLS (1, 2)

****Paternal grandfather had Parkinson’s disease, maternal grandmother had dementia

1. Kosaka T, Fu YJ, Shiga A, Ishidaira H, Tan CF, Tani T, et al. Primary lateral sclerosis: upper-motor-predominant amyotrophic lateral sclerosis with frontotemporal lobar degeneration--immunohistochemical and biochemical analyses of TDP-43. Neuropathology. 2012;32(4):373-84.

2. Mackenzie IRA, Briemberg H. TDP-43 pathology in primary lateral sclerosis. Amyotroph Lateral Scler Frontotemporal Degener. 2020;21(sup1):52-8.

**Supplementary Table 1b. Control Details**

| **No.** | **Unique ID** | **Sex** | **Age**  **at sample collection (y)** | **BMI** | **Highest education** | **Co-morbidities** |
| --- | --- | --- | --- | --- | --- | --- |
| 16 | MND001^#^ | F | 74 | 27.9 | Secondary school | Previous skin cancer |
| 17 | MND002^#^ | M | 70 | 28.4 | Postgraduate | Previous heart attack & heart bypass |
| 18 | MND004 | M | 76 | 24.2 | Undergraduate | Raised cholesterol, hypertension, previous stroke |
| 19 | MND008 | M | 72 | 21.1 | Undergraduate | None |
| 20 | MND010 | M | 78 | 27.8 | Secondary school | Previous heart attack |
| 21 | MND012 | M | 82 | 26.3 | Technical/trade school | Hypertension |
| 22 | MND014^#^ | F | 82 | 30.1 | Technical/trade school | Hypertension and raised cholesterol |
| 23 | MND016^#^ | F | 78 | 19.3 | Undergraduate | Raised cholesterol, previous stroke, previous breast cancer |
| 24 | MND018^#^ | M | 65 | 26.2 | Technical/trade school | None |
| 25 | MND019 | M | 72 | 29.9 | Technical/trade school | None |
| 26 | MND020 | M | 77 | 25.3 | Technical/trade school | Raised cholesterol, hypertension, previous stroke |
| 27 | MND023 | M | 86 | 25.5 | Technical/trade school | Raised cholesterol, hypertension, previous heart attack & heart bypass, previous skin cancer, inflammatory bowel disease |
| 28 | MND024 | F | 74 | 27.4 | Secondary school | Diabetes, raised cholesterol, hypertension, previous heart attack & catheter dilatation in heart |
| 29 | MND027 | F | 86 | 21.9 | Postgraduate | None |
| 30 | MND028^#^ | M | 67 | 28.4 | Secondary school | None |

^#^ Subject sample used for proteome profile array analysis

**Supplementary Table 1c. Metadata of patient and control demographics**

| **General** |  | **M (n)** | **F (n)** | **Mean age ± SD** | **Mean BMI ± SD** |
| --- | --- | --- | --- | --- | --- |
|  | ALS | 10 | 5 | 67.3 ± 10.4 | 25.81 ± 4.64 |
|  | Control | 10 | 5 | 75.9 ± 6.3 years | 25.99 ± 3.07 |
| **Education** |  | **Postgraduate (n)** | **Undergraduate (n)** | **Technical/trade school (n)** | **Secondary school**  **(n)** |
|  | ALS | 2 | 6 | 3 | 4 |
|  | Control | 2 | 3 | 6 | 4 |
| **Co-morbidities** |  | **None** | **Previous heart attack** | **Raised cholesterol** | **Hypertension** |
|  | ALS | 3 | 1 | 7 | 9 |
|  | Control | 5 | 4 | 6 | 6 |
|  |  | **Diabetes** | **Previous stroke** | **Cancer** | **Other** |
|  | ALS | 2 | 0 | 1 | 1 |
|  | Control | 1 | 3 | 3 | 1 |

**Supplementary Table 2. Cytokine Bead Array Flex-Sets Used**

| **Cytokine detection** | **Bead position** | **Species** | **Supplier** | **Catalogue number** |
| --- | --- | --- | --- | --- |
| IL-6 | A7 | Human | BD Biosciences | 558276 |
| IL-8 | A9 | Human | BD Biosciences | 558277 |
| RANTES | D4 | Human | BD Biosciences | 558324 |
| MCP-1 | D8 | Human | BD Biosciences | 558287 |
| IP-10 | B5 | Human | BD Biosciences | 558280 |
| Fractalkine | C6 | Human | BD Biosciences | 560265 |
| sICAM-1 | A4 | Human | BD Biosciences | 560269 |
| sVCAM-1 | D6 | Human | BD Biosciences | 560427 |

**Supplementary Table 3. Cytokine Panel in Proteome Profiler Human XL Cytokine Array Kit (#ARY022B)**

| Adiponectin/Acrp30 | Interferon (IFN)-gamma | C–C motif chemokine ligand 2 (CCL2)/ Monocyte chemoattractant protein-1 (MCP-1) |
| --- | --- | --- |
| Angiogenin | Insulin-like growth factor binding protein 2 (IGFBP-2) | C–C motif chemokine ligand 7 **(**CCL7**)**/ Monocyte chemoattractant protein-3 **(**MCP-3**)** |
| Angiopoietin-1 | Insulin-like growth factor binding protein 2 (IGFBP-3) | Macrophage colony-stimulating factor (M-CSF) |
| Angiopoietin-2 | Interleukin-1 alpha (IL-1 alpha)/ Interleukin-1F1 (IL-1F1) | Macrophage migration inhibitory factor (MIF) |
| Apolipoprotein A1 | Interleukin-1 beta (IL-1 beta)/ Interleukin-1F2 (IL-1F2) | C-X-C motif ligand 9 (CXCL9)/ Monokine induced by gamma interferon (MIG) |
| B-cell activating factor (BAFF)/ B Lymphocyte Stimulator (BLyS)/ Tumor Necrosis Factor Superfamily Member 13B (TNFSF13B) | Interleukin 1 receptor antagonist (IL-1RA)/ Interleukin-1F3 (IL-1F3) | C–C motif chemokine ligand 3 (CCL3)/ C–C motif chemokine ligand 4 (CCL4)/ Macrophage inflammatory protein (MIP)-1 alpha/beta |
| Brain-derived neurotrophic factor (BDNF) | Interleukin-2 (IL-2) | C–C motif chemokine ligand 20 (CCL20)/ Macrophage inflammatory protein (MIP)-3 alpha |
| Cluster of differentiation 14 (CD14) | Interleukin-3 (IL-3) | C–C motif chemokine ligand 19 (CCL19)/ Macrophage inflammatory protein (MIP)-3 beta |
| Cluster of differentiation 30 (CD30) | Interleukin-4 (IL-4) | Matrix metalloproteinase 9 (MMP-9) |
| Cluster of differentiation 31 (CD31)/ Platelet endothelial cell adhesion molecule-1 (PECAM-1) | Interleukin-5 (IL-5) | Myeloperoxidase |
| Cluster of differentiation 40 (CD40) Ligand/ Tumor Necrosis Factor (Ligand) Superfamily Member 5 (TNFSF5) | Interleukin-6 (IL-6) | Osteopontin (OPN)/ Secreted Phosphoprotein 1 (SPP1) |
| Chitinase 3-like | Interleukin-8 (IL-8) | Platelet-derived growth factor (PDGF)-AA |
| Complement Component C5/C5a | Interleukin-10 (IL-10) | Platelet-derived growth factor (PDGF)-AB/BB |
| Complement Factor D | Interleukin-11 (IL-11) | Pentraxin 3/ Tumor Necrosis Factor-stimulated gene 14 (TSG-14) |
| C-Reactive Protein (CRP) | Interleukin-12 (IL-12) p70 | C-X-C Motif Chemokine Ligand 4 (CXCL4)/ Platelet factor 4 (PF4) |
| Cripto-1 | Interleukin-13 (IL-13) | Receptor for advanced glycation end products (RAGE) |
| Cystatin C | Interleukin-15 (IL-15) | C–C motif chemokine ligand 5 (CCL5)/ Regulated upon Activation, Normal T Cell Expressed and Presumably Secreted (RANTES) |
| Dickkopf WNT signaling pathway inhibitor 1 (DKK-1) | Interleukin-16 (IL-16) | Retinol binding protein 4 (RBP4) |
| Dipeptidyl peptidase IV (DPPIV)/ Cluster of differentiation 26 (CD26) | Interleukin-17A (IL-17A) | Relaxin-2 |
| Epidermal growth factor (EGF) | Interleukin-18 Binding Protein a (IL-18 BPa) | Resistin |
| C-X-C Motif Chemokine Ligand 5 (CXCL5)/ Epithelial-derived neutrophil-activating peptide 78 (ENA-78) | Interleukin-19 (IL-19) | C-X-C Motif Chemokine Ligand 12 (CXCL12)/ Stromal cell-derived factor (SDF)-1 alpha |
| Endoglin/ Cluster of differentiation 105 (CD105) | Interleukin-22 (IL-22) | Serpin E1/ Plasminogen activator inhibitor-1 (PAI-1) |
| Extracellular matrix metalloproteinase inducer (EMMPRIN)/ Cluster of differentiation 147 (CD147) | Interleukin-23 (IL-23) | Sex hormone-binding globulin (SHBG) |
| Fas Ligand | Interleukin-24 (IL-24) | Suppression Of Tumorigenicity 2 (ST2)/ Interleukin-1 Receptor 4 (IL1 R4) |
| Fibroblast growth factor (FGF) basic | Interleukin-27 (IL-27) | C–C motif chemokine ligand 17 (CCL17)/ Thymus- and activation-regulated chemokine (TARC) |
| Keratinocyte growth factor (KGF)/ Fibroblast growth factor 7 (FGF-7) | Interleukin-31 (IL-31) | Trefoil Factor 3 (TFF3) |
| Fibroblast growth factor 19 (FGF-19) | Interleukin-32 (IL-32) alpha/beta/gamma | Transferrin receptor (TfR) |
| Fms-Like Tyrosine Kinase 3 (FLT-3) Ligand | Interleukin-33 (IL-33) | Transforming growth factor (TGF)-alpha |
| Granulocyte colony stimulating factor (G-CSF) | Interleukin-34 (IL-34) | Thrombospondin-1 |
| Growth/Differentiation Factor-15 (GDF-15) | C-X-C motif chemokine ligand 10 (CXCL10)/ Interferon gamma-induced protein 10 (IP-10) | T-cell immunoglobulin 1 (TIM-1) |
| Granulocyte-macrophage colony-stimulating factor (GM-CSF) | C-X-C motif chemokine 11 (CXCL11)/ Interferon-inducible T-cell alpha chemoattractant (I-TAC) | Tumour Necrosis Factor (TNF)-alpha |
| C-X-C Motif Chemokine Ligand 1 (CXCL1)/ Growth-regulated oncogene (GRO) alpha | Kallikrein 3/ Prostate Specific Antigen (PSA) | Urokinase-type plasminogen activator receptor (uPAR) |
| Growth Hormone (GH) | Leptin | Vascular cell adhesion molecule 1 (VCAM-1) |
| Hepatocyte Growth Factor (HGF) | Leukemia inhibitory factor (LIF) | Vascular endothelial growth factor (VEGF) |
| Intercellular Adhesion Molecule 1 (ICAM-1)/ Cluster of differentiation 54 (CD54) | Lipocalin-2/ Neutrophil gelatinase-associated lipocalin (NGAL) | Vitamin D binding protein |

**Supplementary Table 4. Antibodies Used**

| **Antibody** | **Type** | **Supplier** | **Catalogue number** | **Dilution** | **Species** |
| --- | --- | --- | --- | --- | --- |
| Anti-MCP1 | Primary | BioLegend | 502607 | 1:500 | Mouse |
| Anti-VCAM1 | Primary | Abcam | Ab134047 | 1:500 | Rabbit |
| Alexa Fluor® 594 anti-mouse | Secondary | Invitrogen | A32744 | 1:500 | Donkey |
| Alexa Fluor® 488 anti-rabbit | Secondary | Invitrogen | A32790 | 1:500 | Donkey |

**Supplementary Figure 1:** *Optimization of serum dilutions*

**
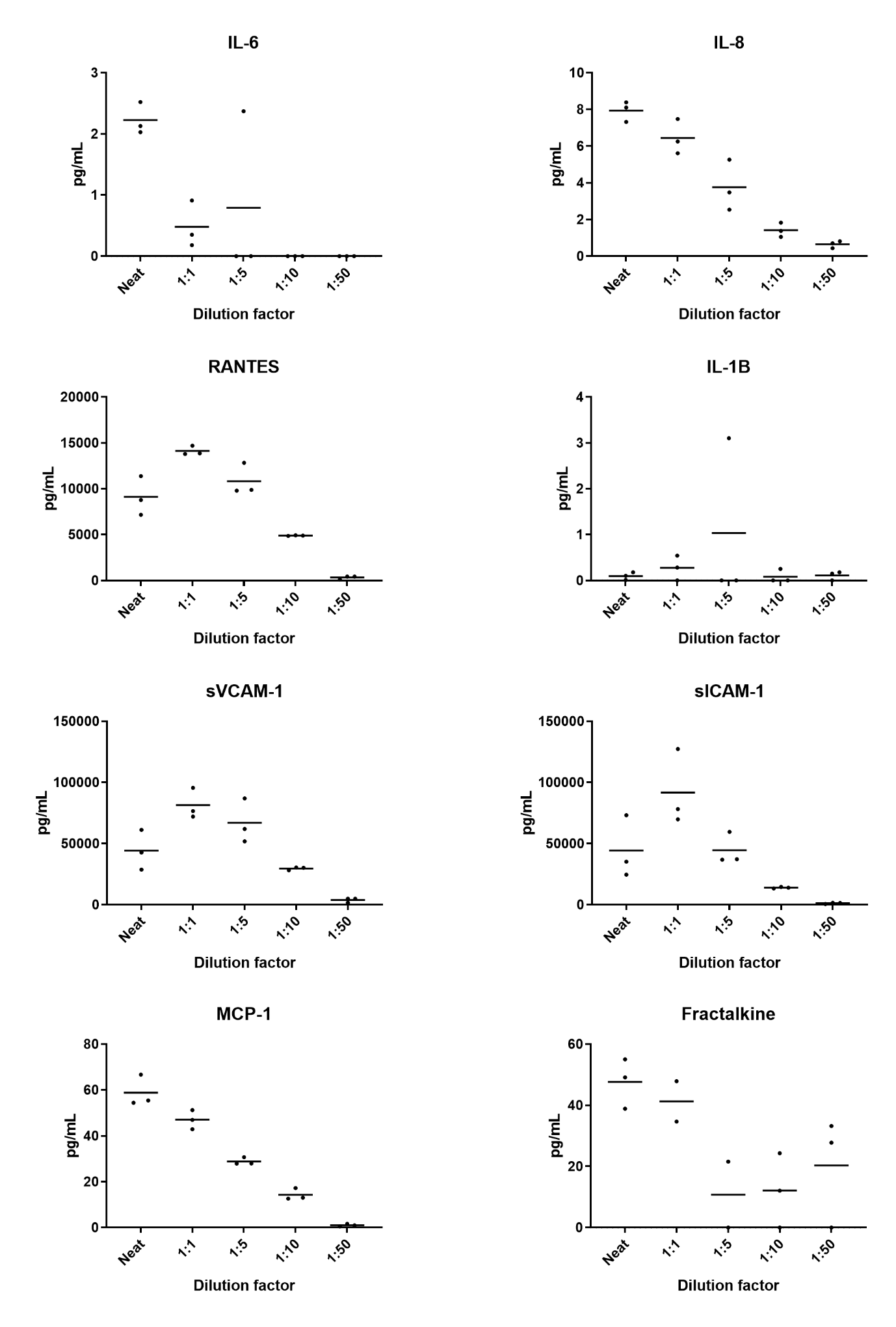
**

**Supplementary Figure 2:** *Serum cytokine levels by subject age*


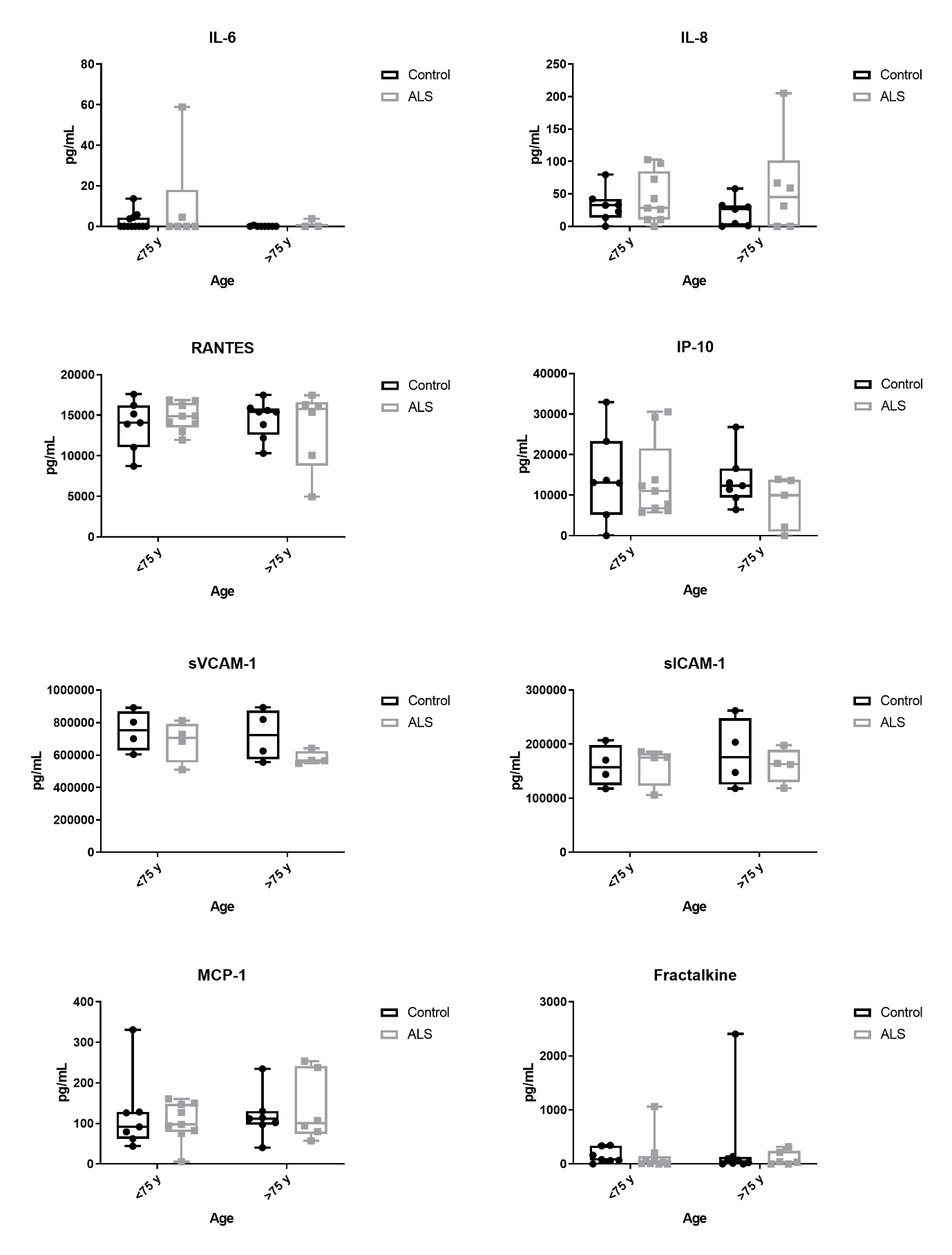


**Supplementary Figure 3:** *Serum cytokine levels by disease duration (ALS subjects only)*


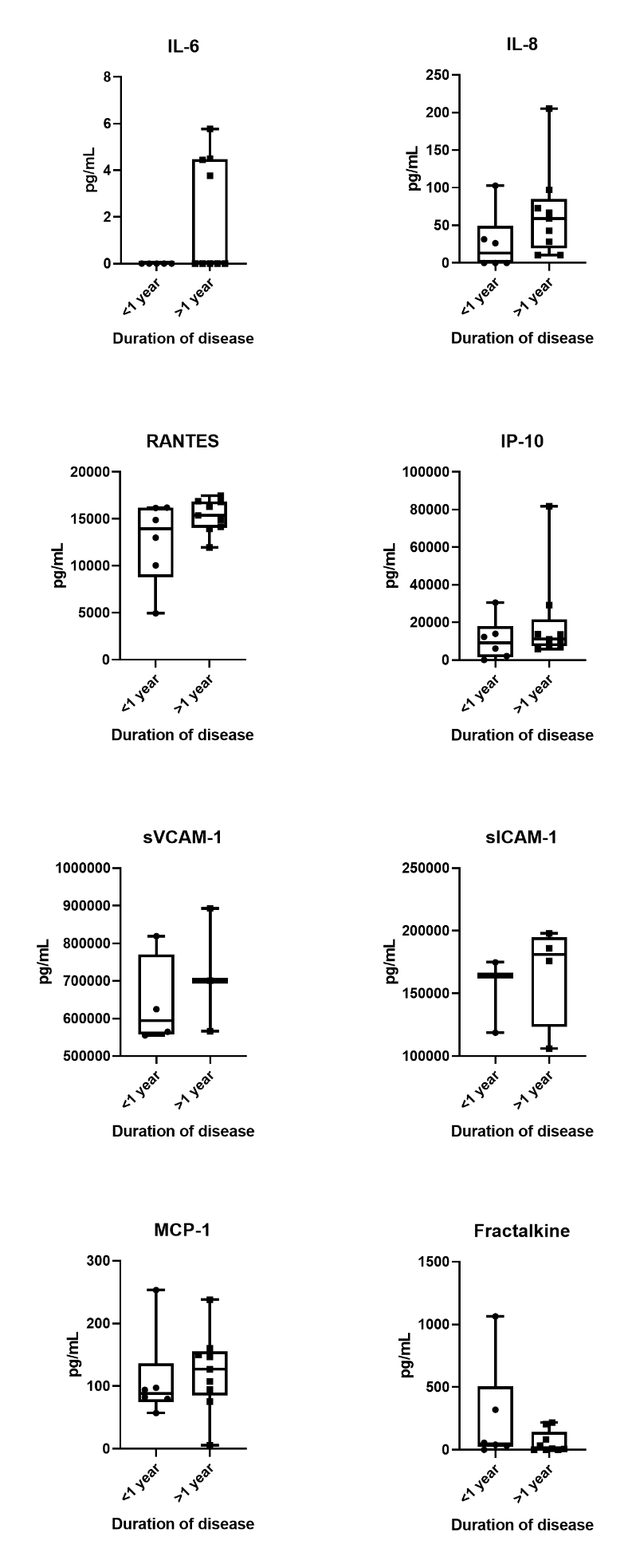


**Supplementary Figure 4:** *S100β ELISA assay standard curve*

**
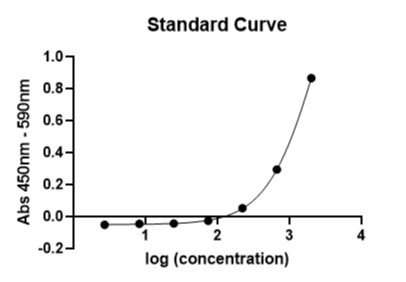
**

**Supplementary Figure 5:** *PCA of ALS and control serum proteome profiles*

**
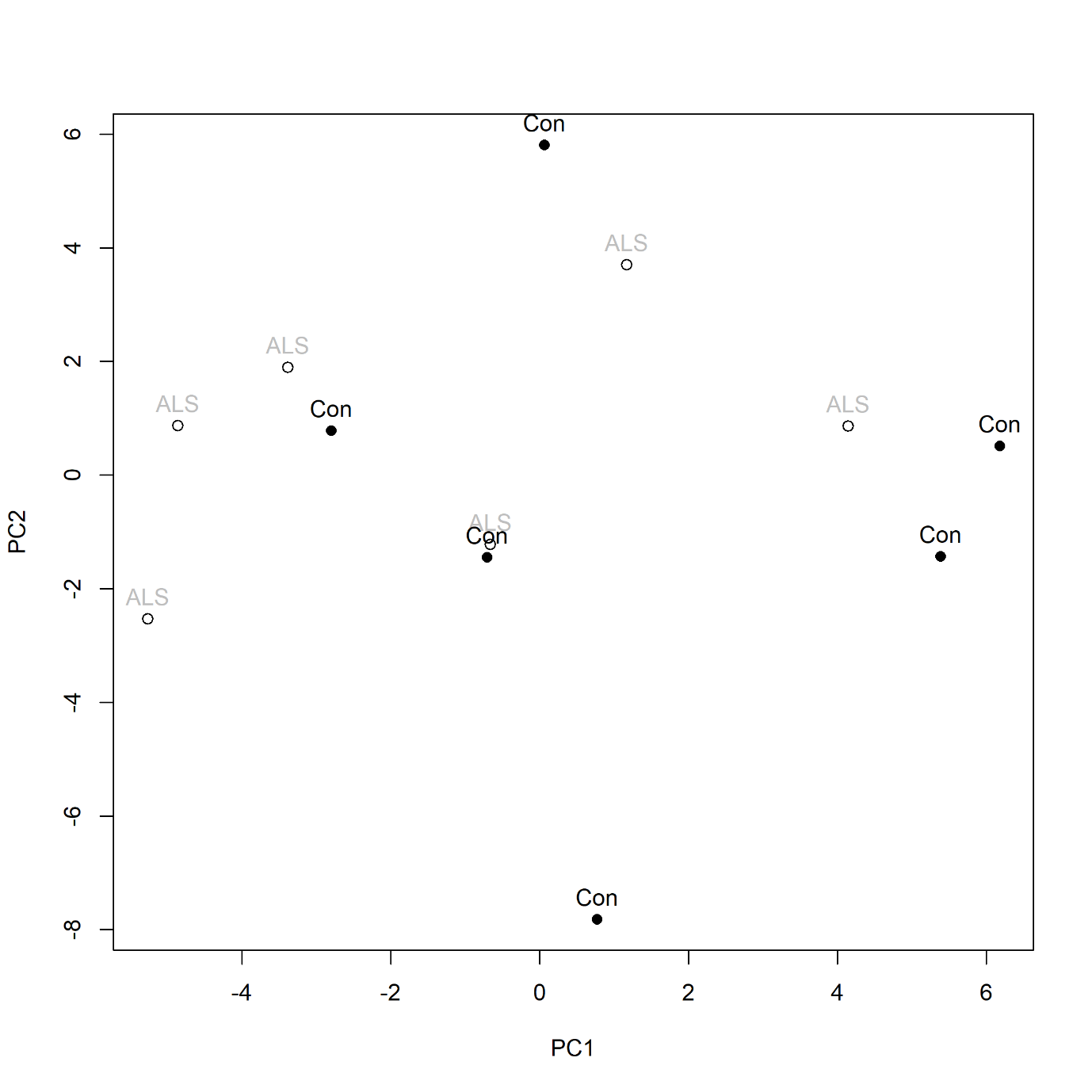
**
